# Low-Dose Clozapine Reduces Aggression in Violent Substance Use Disorder Patients at a Secure Rehabilitation Center

**DOI:** 10.1155/crps/9578923

**Published:** 2025-10-03

**Authors:** Suhair Mohammed Yousuf, Abdelrahman Zohir Khaled Eldous, Faycal Walid Ikhlef, Nirvana Swamy Kudlur Chandrappa, Majid Ali Y. A. Al Abdulla

**Affiliations:** Mental Health Services, Hamad Medical Corporation, Doha, Qatar

**Keywords:** aggressive behavior, clozapine, comorbid psychiatric conditions, substance use disorder

## Abstract

Managing aggressive behaviors in patients with substance use disorders (SUDs), particularly when accompanied by comorbid psychiatric disorders, presents considerable challenges. Clozapine is an atypical antipsychotic known for its effectiveness in treatment-resistant schizophrenia with evidence in support of its ability to reduce violent and aggressive behaviors. This case report describes two patients who were treated with clozapine and showed reduction in violent behaviors, as well as improvement in addiction issues during their stay in a secure rehabilitation center. This observation suggests that clozapine may offer considerable benefits in managing aggression in SUD patients with co-occurring psychiatric illness and highlights the need for further research to assess its broader applicability in complex cases beyond treatment resistant schizophrenia.

## 1. Introduction

### 1.1. Background

Aggressive behavior in patients with substance use disorder (SUD) poses significant challenges in treatment and management, particularly when compounded by comorbid psychiatric disorders. Substance abuse not only exacerbates underlying psychiatric disorders but also contributes to the development of others. Traditional treatments, including regular antipsychotics and psychotherapeutic interventions, often fall short in managing aggression within this population, highlighting the need for alternative therapeutic options. Clozapine, an atypical antipsychotic, has demonstrated effectiveness in reducing aggression in treatment-resistant schizophrenia and other psychiatric conditions [1]. However, its use in patients with SUD remains underexplored, despite its unique ability to modulate dopaminergic, serotonergic, and glutamatergic systems. This case series investigates the potential of clozapine in management of SUD patients, a growing concern given the complexity of dual diagnosis.

### 1.2. Methods

In this report, we present two cases from Umm Slal Treatment and Rehabilitation Center (USTRC) in Doha, Qatar. USTRC treats patients with SUD and comorbid psychiatric conditions who exhibit significant aggression, addiction to substances, and psychiatric disorder. Aggression is measured by the Dynamic Appraisal of Situational Aggression (DASA) [2] which is administered by trained nursing staff. DASA is a seven-item observer-rated tool, which assess the likelihood of imminent aggression within 24 h. In the inpatient version, scoring is made in two steps: First assessing the risk of aggression and then, recording the aggression. Scoring criteria is made from 0 to 1 for irritability, impulsivity, unwillingness to follow directions, sensitivity to perceived provocation, easily angered when requests are denied, negative attitudes, and verbal threats, revealing a maximum score of seven for recorded aggression. DASA scores of 0–1, 2–3, and >3 is interpreted as low, moderate, and high aggression risk, respectively (Table 1). DASA is available within Cerner, the facility's internal electronic medical record (EMR) system. The selection of DASA as the sole instrument in this study was based on institutional protocols and the availability of retrospective data. As the healthcare facility's formally mandated risk assessment tool for aggression, the DASA is routinely utilized in inpatient settings for standardized daily documentation. Furthermore, it was the only aggression scale consistently documented for all patients as part of routine clinical practice, ensuring comprehensive and systematic data capture. A retrospective review of case notes was conducted, utilizing DASA, to assessing the likelihood of imminent aggression within 24 h. Patients were considered for clozapine therapy if they were diagnosed with SUD with comorbid severe behavioral dysregulation, including recurrent violent episodes, that is, three or more documented incidents each week in the preceding 4 weeks. In addition, the patients should have failed to respond to at least two antipsychotics, not adequately improved despite trials of adjunctive medications, for example, mood stabilizers, or benzodiazepines and structured nonpharmacological interventions, for example, cognitive behavioral therapy. Furthermore, the patients should have displayed persistent aggression, defined as a DASA score of three or more for at least two consecutive weeks. Patients were excluded if they presented with acute intoxication or active withdrawal at the time of evaluation or had absolute contraindications to clozapine. All clinical diagnoses and exclusions were determined by the treating psychiatrist according to the criteria outlined in the International Statistical Classification of Diseases and Related Health Problems, 11th edition (ICD-11) and the Diagnostic and Statistical Manual of Mental Disorders, 5th edition (DSM-5).

### 1.3. Aims

The primary aim of this report was to evaluate clozapine's efficacy in reducing aggressive behavior, achieved by comparing 24 h daily DASA scores 2 weeks before and 3 weeks after clozapine administration. Key objectives included assessing baseline aggression levels, monitoring changes postclozapine initiation, evaluating safety and tolerability, and documenting side effects. Additionally, the study aimed to assess improvements in patients' quality of life and overall functioning.

## 2. Case Presentation

### 2.1. Case 1

Patient A was a married female educator in her mid-30s with a long-standing history of borderline personality disorder and ketamine dependence who was admitted to USTRC. Patient A had been under psychiatric care for 9 years when admitted to USTRC. The treatment has been characterized by poor medication adherence and inconsistent engagement. Despite trials of various antidepressants, mood stabilizers, and psychological treatments, Patient A had experienced frequent crises with presentations to psychiatric services and emergency departments with agitation, insomnia, anxiety, and mood problems triggered by family or marital conflicts. Patient A demonstrated a repeated pattern of seeking sedatives, specifically ketamine, pregabalin, and diazepam, with repeated frequent contact with emergency services multiple times every month. During mental health hospital admissions, Patient A exhibited significant self-harm and aggressive behaviors towards herself and staff, primarily driven by drug-seeking behavior. This necessitated the repeated use of tranquilizing medications such as lorazepam, haloperidol, chlorpromazine, olanzapine, and diphenhydramine.

#### 2.1.1. Hospital Course

Patient A was transferred to our secure rehabilitation unit approximately 2 weeks after her admission to a mental health inpatient unit for further management of her substance use and severe aggression, which included daily property damage and physical aggression towards staff. Upon admission, urine toxicology screening was negative. During the first week, Patient A exhibited severe violent behavior and repeatedly requested ketamine. Mechanical restraints were required on four occasions after verbal de-escalation and oral medications, including antipsychotics and benzodiazepines failed to manage her agitation. Pro re nata (PRN) haloperidol and lorazepam were administered during these episodes, and Patient A's observation level was raised to 2:1, that is two staff members were allocated for intensive nursing care. Patient A's regimen was adjusted to include haloperidol, chlorpromazine, and procyclidine, with minimal improvement.

In the second week, a fifth restraint was necessary, following which long-acting injectable (LAI) zuclopenthixol decanoate was initiated and titrated to 200 mg IM weekly; however, her response remained poor in the initial weeks.

In this context, potential clinical confounders such as prior ketamine use and delayed initiation of zuclopenthixol were considered. The patient had received a single dose of ketamine approximately 3 weeks before admission to our facility, by which time acute pharmacologic effects would have subsided. No signs of withdrawal were noted and formal withdrawal scales were not indicated. Notably, zuclopenthixol LAI was introduced 6 weeks into her stay, after continued behavioral dysregulation. Therefore, the early signs of engagement and behavioral stabilization observed prior to the antipsychotic effect are more likely attributable to sustained abstinence, therapeutic containment, and structured psychosocial intervention.

#### 2.1.2. Clozapine Initiation

Following a multidisciplinary team meeting and case conference on risk management, it was decided to add clozapine to the patient's medication regimen. Before initiation, the patient and family were engaged in discussion about risks and benefits of the medication and consented to close monitoring, including weekly laboratory tests, electroencephalograms (ECGs), and metabolic check-ups. Thus, baseline laboratory tests and an ECG were conducted prior to clozapine initiation, followed by weekly laboratory monitoring postinitiation. Clozapine was initiated at a dose of 25 mg/day and gradually titrated to a maintenance dose of 100 mg daily. The notion of a standardized clozapine titration protocol is clinically variable due to the substantial variability in patient-specific factors and clinical profiles [3, 4]. For instance, younger patients with severe aggression or acute symptomatology may tolerate relatively rapid titration, particularly in inpatient settings where close monitoring of vital signs and immediate detection of adverse effects are feasible [1, 2]. While institutional guidelines and published literature provide general titration frameworks [5], these must be tailored to individual patient needs, balancing efficacy, and tolerability. However, adjustments may be necessary based on clinical response and side-effect profiles [3, 4].

During the initial phase, clozapine was coadministered with LAI zuclopenthixol decanoate 200 mg intramuscularly weekly. However, zuclopenthixol was discontinued by the second week to minimize potential adverse effects.

#### 2.1.3. Laboratory Findings and Side Effects

After starting clozapine, the patient reported experiencing an “electric shock feeling” and was observed to have resting tachycardia at a daily dose of 100 mg. Heart rate recordings during this period were noted to be 101, 130, 121, 123, and 126 beats per minute. With continued monitoring and appropriate management, the heart rate gradually decreased over time.

An ECG showed no abnormalities and the QTc interval remained within normal limits. An internal medicine consult was conducted, and propranolol 40 mg four times a day was added to the regimen as a trial to manage the tachycardia. However, clozapine is associated with severe complications, including myocarditis and cardiomyopathy [6], warranting enhanced cardiac monitoring. Weekly ECG monitoring was performed for 6 weeks postinitiation, with all QTc intervals remaining below 450 ms. Concurrent weekly assessments of high-sensitivity troponin and C-reactive protein levels consistently were within normal reference ranges.

#### 2.1.4. Follow-Up and Outcomes

**Figure 1:**
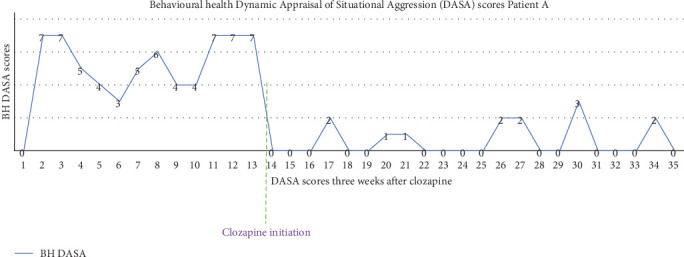
Displays the daily 24-h recorded Dynamic Appraisal of Situational Aggression (DASA) scores for Case 1. The graph illustrates a significant improvement in scores following the initiation of clozapine therapy.

Before beginning clozapine therapy, Patient A's DASA levels were recorded as exceeding three, with elevated scores in irritability, impulsivity, noncompliance, and verbal threats, as illustrated in Figure 1. Three weeks into the therapy, follow-up DASA scores dropped to zero, indicating a low risk. Patient A became more cooperative with the treatment plan and actively participated in biopsychosocial therapeutic sessions with an occupational therapist, a psychologist, and a physical therapist. A trial of permission with family received favorable feedback, leading to her successful discharge after completing the 3-month program. During Patient A's inpatient stay, zuclopenthixol was discontinued and her at-home medications were adjusted to include clozapine 100 mg daily, aripiprazole 7.5 mg daily, and propranolol 20 mg three times a day.

The clozapine 100 mg daily dose was retained as it achieved the primary outcome, that is, aggression reduction and further dose escalation was deemed unnecessary considering both therapeutic response and emerging side effects.

When on clozapine therapy for 8 months, Patient A demonstrated sustained clinical stability throughout the first 6-month outpatient follow-up period, adhering to a structured monitoring protocol involving weekly clinical assessments. As part of a comprehensive SUD management plan, random urine drug screenings were conducted periodically, all of which yielded negative results, indicating maintained abstinence from illicit substances Patient A's outpatient weekly laboratory investigations were generally within normal limits. However, mildly elevated liver enzymes were noted, with an alanine aminotransferase (ALT) of 44 U/L (reference range: 7–56 U/L) and an aspartate aminotransferase (AST) of 74 U/L (reference range: 5–40 U/L), which were attributed to clozapine use. The patient then transitioned to monthly appointments with good engagement with case manager/care coordinator, psychiatrist, and psychologist. Patient A reported no cravings for ketamine or other substances, had no other active medical concerns, and had successfully resumed her role as an educator while maintaining strong engagement with her family and community. Moreover, this extended period of follow-up revealed no episodes of violence or aggression. This clinical observation was further corroborated by collateral reports from family members, who consistently denied witnessing any concerning behavioral disturbances in home and community settings.

### 2.2. Case 2

Patient B was a 30-year-old single male government officer, admitted to USTRC for poly-SUD, with alcohol, methamphetamine, and cannabis as his primary substances of abuse. He had previously sought treatment at a private center for 3 months last year, but experienced a relapse.

#### 2.2.1. Hospital Course

Upon admission, a urine toxicology test was conducted, with negative results. Patient B successfully completed 2 weeks of detoxification and was subsequently transferred to the rehabilitation phase. His medication regimen included acamprosate 333 mg three times a day and quetiapine 200 mg at bedtime. A psychological assessment using the Personality Inventory for DSM-5 (Brief Form, Adult) revealed an overall score of 24 out of 75, indicating “low intensity” of personality dysfunction [7]. Patient B scored moderately on the “Antagonism Trait” with a score of 10 out of 15, reflecting callousness, combativeness, and grandiosity, which correlates with externalizing behaviors such as antisocial behavior, aggression, and substance use. The patient scored 24 out of 75 on the overall Personality Inventory, indicating “low intensity” of personality dysfunction. He scored 10 out of 15 on the Antagonism domain, reflecting moderate traits of callousness and combativeness, which correlate with externalizing behaviors such as antisocial behavior, aggression, and substance use. Scores for the remaining domains—Negative Affect, Detachment, Disinhibition, and Psychoticism—were very low, indicating minimal presence of traits associated with these dimensions.

During the rehabilitation phase, Patient B exhibited fluctuating mood, emotional dysregulation, and poor engagement in therapeutic sessions. He was observed to be impulsive and aggressive towards staff and security personnel, resulting in a mechanical restraint. He received zuclopenthixol acetate 50 mg once during restraint. Following the event, Patient B was transferred to detoxification and his observation level was increased to 1:1 continuous nursing care, that is, a designated staff member continuously monitored him. Patient B's daily DASA scores peaked during the detoxification phase, reaching a maximum score of 7 on the fourth day of his transfer to the detoxification unit. Patient B continued to have aggressive episodes in the unit. His updated medication regimen included LAI zuclopenthixol decanoate 150 mg weekly and mirtazapine 15 mg/day.

#### 2.2.2. Clozapine Initiation

Following a multidisciplinary team meeting, it was decided to introduce clozapine to the patient's medication regimen. Clozapine was initiated at 25 mg/day and titrated up to a maintenance dose of 200 mg/day, in combination with zuclopenthixol decanoate 200 mg IM weekly. Baseline laboratory tests and an ECG were conducted prior to clozapine initiation, followed by weekly laboratory monitoring post-initiation. Following the initiation of clozapine, Patient B's DASA scores decreased to 3 by the third day of the treatment.

#### 2.2.3. Clozapine Initiation

Following a multidisciplinary team meeting, it was decided to introduce clozapine to the patient's medication regimen. Clozapine was initiated at 25 mg and titrated up to a maintenance dose of 200 mg, in combination with zuclopenthixol 200 mg IM weekly. Baseline laboratory tests and an ECG were conducted prior to clozapine initiation, followed by weekly lab monitoring postinitiation. Following the initiation of clozapine, his DASA scores decreased to 3 by the third day of the treatment.

#### 2.2.4. Lab Findings and Side Effects

Baseline laboratory tests and an ECG were performed before initiating clozapine, all of which were normal. No significant side effects were reported following the initiation of clozapine.

#### 2.2.5. Follow-Up and Outcomes

**Figure 2:**
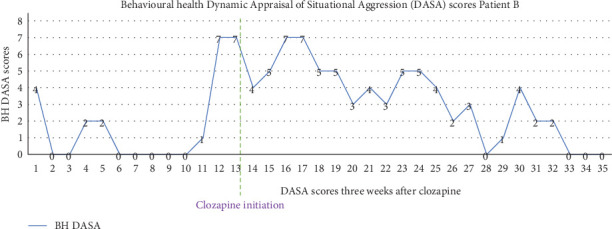
Displays the daily 24-h recorded Dynamic Appraisal of Situational Aggression (DASA) scores for Case 2. The graph illustrates a steady decline in scores following the initiation of clozapine therapy.

Prior to the initiation of clozapine therapy, Patient B's DASA scores consistently exceeding a threshold of 3, indicative of heightened risk factors including irritability, impulsivity, noncompliance, verbal threats, and a negative attitude, as illustrated in Figure 2. On the day of clozapine initiation, the DASA score escalated to a maximum recorded value of 7, reflecting a critical peak in situational aggression risk. Following 3 weeks of clozapine therapy, subsequent DASA assessments demonstrated a marked reduction, with daily scores stabilizing at zero, suggesting a significant amelioration of aggressive tendencies. Patient B was also observed to be adjusting well and actively participating in individual therapeutic sessions and was transferred back to the rehabilitation phase. Upon completing the 3-month program, Patient B was successfully discharged on a regimen of clozapine 75 mg in the morning and 125 mg at bedtime. All his other medications were stopped. Patient B attended the outpatient clinic once, but later disengaged from the services. Notably, the single follow-up visits and the early disengagement from aftercare represented a significant limitation in our ability to evaluate treatment outcomes.

### 2.3. Summary Table

See Table 2 for a summary of patient characteristics, treatments, and outcomes.

## 3. Results

See Figures 1 and 2.

## 4. Discussion

We present the cases of two patients with SUD and severe aggression, who were treated with clozapine with substantial reduction of both aggression and substance use. None of the patients reported in this case study had histories of epilepsy, brain injury, or learning disorders or had a concurrent diagnosis of schizophrenia or schizoaffective disorder. Thus, clozapine was prescribed off label. Clozapine's unique antipsychotic profile is partly attributed to its high-affinity antagonism at serotonin 5-HT_2_A and 5-HT_2_C receptors [8], which is believed to modulate dopaminergic activity and reduce both positive and negative symptoms of schizophrenia [9]. Its efficacy extends beyond psychosis management to ameliorate frequently co-occurring symptoms such as anxiety, insomnia, and affective instability, which often complicate SUD treatment and recovery [10–12]. Notably, clozapine's antiaggressive effects may involve serotonin-mediated reduction in impulsivity [13], dual modulation of dopamine–glutamate pathways [14] and broader “aggressivolytic” properties in complex neuropsychiatric conditions [15].

Following our narrative, both patients exhibited significant clinical improvement after the initiation of clozapine treatment, particularly in reducing aggression and violence risk. A concise comparison of demographics, substance use profiles, treatment regimens, and short-term outcomes is presented in Table 2. Notable improvements were observed in cognitive-perceptual and emotional regulation, with the most pronounced effects seen in impulsive-behavioral symptoms. Clozapine demonstrated efficacy across all symptom domains [16], with the greatest benefit seen in the reduction of impulsivity and anger [17]. The structured rehabilitation environment likely contributed to the overall management of aggression in both cases, but the most significant reduction in aggressive behaviors correlated temporally with the initiation of clozapine, suggesting that the pharmacological changes played the decisive role. For Case 1, despite being in a controlled setting with 2:1 intense observation and a structured routine the DASA scores remained high from the time of transfer from the acute psychiatry unit through admission to our facility as shown in Figure 1. This persistence of aggression indicates that environmental modifications alone were insufficient to produce meaningful behavioral change. However, once clozapine was introduced, a notable decline in aggression was observed, likely targeted the underlying neurobiological drivers of aggression more effectively than environmental adjustments alone. Thus, while the rehabilitation setting provided necessary support and monitoring, the timing of improvement strongly implicates clozapine as the key intervention responsible for the reduction in aggression. This aligns with evidence supporting clozapine's effectiveness in managing symptoms such as hostile agitation and assaultive violence.

Some literature supports clozapine's effectiveness in treating aggressive behavior, particularly in patients with schizophrenia and comorbid SUDs [5]. While studies of antipsychotics in community settings have shown clozapine's superior efficacy over other agents in reducing violence, these findings are consistent with our observations [18]. The presence of comorbid SUDs complicates the clinical picture, as such conditions are known risk factors for increased violence, nonadherence, and recidivism [18, 19]. Growing clinical evidence highlights the therapeutic potential of second-generation antipsychotics (SGAs) in managing dual diagnosis patients, where psychotic symptoms intersect with substance use. SGAs, with their unique receptor profiles targeting dopamine, serotonin, and other neurotransmitter systems, offer advantages in addressing core psychiatric symptoms and mitigating factors that perpetuate addiction [10]. Their efficacy extends beyond psychosis management to ameliorate frequently co-occurring symptoms such as anxiety, insomnia, and affective instability, which often complicate SUD treatment and recovery [20].

Despite its proven efficacy in treating treatment-resistant schizophrenia, clozapine remains underutilized, likely due to barriers such as prescriber hesitancy, concerns about side effects, and the need for frequent and complex haematological monitoring.

Oral clozapine offers unique advantages over other oral antipsychotics in such cases [18, 19]. Additionally, LAIs, though not including clozapine, have been shown to improve medication adherence and reduce relapse rates compared to oral formulations. LAIs also facilitate the implementation of functional skills training, which can enhance patients' ability to live independently and interact appropriately [19]. Clozapine may help reduce substance use severity [21], though this requires further investigation. The goal is that by reducing violent behavior, clozapine may improve overall treatment outcomes, enhance communication and compliance, and support better engagement in SUD treatment [17].

The relationship between clozapine and SUDs is particularly noteworthy. Anecdotal evidence suggests that clozapine may also help to reduce the severity of substance use in psychiatric patients [22], although this area requires further investigation. Despite the generally poor prognosis for patients with schizophrenia and comorbid SUDs, clozapine has shown a greater antiaggressive effect, particularly in patients with conduct disorder, compared to haloperidol [21, 23]. This reinforces the findings that clozapine can be substantially more effective in managing aggression in specific patient populations [16]. While standard clozapine dosing for treatment-resistant schizophrenia typically ranges from 200 to 450 mg daily [24], significant reductions in aggression have been observed at lower doses (25–100 mg/day) in diverse populations, including patients with borderline personality disorder [25], treatment-refractory aggression [26], comorbid obsessive compulsive disorder (OCD) [27], or learning disabilities [28]. This study has several limitations that warrant careful consideration when interpreting the results. First, the retrospective design relying on clinical chart reviews which introduces potential biases, including incomplete documentation and variability in how aggression episodes were recorded. Given the absence of a control group or comparison to alternative treatments, we cannot definitively attribute the observed reductions in aggression solely to clozapine, as other therapeutic interventions may have contributed.

Additionally, the small sample size and highly specific patient selection with treatment-resistant aggression and prior antipsychotic failure limit the generalizability of our findings. These patients may not represent the broader population with SUD and comorbid aggression. Furthermore, the DASA assessments were conducted by unblinded treating staff, which may have introduced observer bias, particularly given the known antiaggressive effects of clozapine.

Other confounding factors include the structured rehabilitation environment, concomitant medications, and variability in therapeutic relationships between patients and staff, all of which could have influenced outcomes independently of clozapine. The almost immediate favorable response to clozapine in Case 1 may also be explained by abatement of the patient's ketamine withdrawal syndrome and the combined effect of clozapine and the previously given LAI antipsychotic zuclopenthixol. The short follow-up period, especially in Case 2, further restricts our ability to assess long-term efficacy and relapse prevention.

Finally, the lack of standardized craving assessments or clozapine plasma level monitoring means we cannot fully evaluate its impact on substance use or confirm optimal dosing. Future prospective studies with larger samples and controlled comparisons are needed to validate these preliminary findings. It is important to note that these findings are hypothesis-generating and underscore the need for well-designed and prospective controlled studies to further explore the role of clozapine in managing aggression within this patient population. A further complicating factor in managing aggression and SUDs is the increasing prevalence of novel psychoactive substances (NPSs), which pose significant clinical and safety challenges. NPS use has been strongly associated with unpredictable psychiatric presentations, suicidality, and heightened aggression, particularly in high-risk environments such as prisons or custodial settings [29, 30]. Their pharmacological variability, rapid emergence, and legal ambiguity make them difficult to detect and manage using traditional protocols. Recent systematic reviews highlight concerning trends, including the misuse of over-the-counter drugs, prescription-only medications, and newly synthesized compounds that can mimic or amplify psychosis-like states and disrupt recovery trajectories [31]. These findings underline the importance of screening for NPS use and tailoring treatment strategies accordingly in patients with comorbid aggression and substance misuse.

In addition, emerging evidence supports the potential of psychedelic therapy in the treatment of SUDs. Compounds such as ibogaine and noribogaine have shown promising results in reducing withdrawal symptoms and substance cravings, potentially facilitating sustained recovery through neurochemical and experiential mechanisms [32]. Furthermore, clinical trials evaluating psychedelic-assisted psychotherapy—including substances like psilocybin and 3,4-methylenedioxymethamphetamine (MDMA) have reported improved abstinence rates, emotional regulation, and insight-driven behavior change among SUD populations [33]. While still experimental and requiring rigorous validation, these novel approaches may complement traditional pharmacotherapy in the future and should be considered in the evolving landscape of integrated addiction treatment.

Overall, the literature supports the use of antipsychotics, with clozapine being particularly effective, in reducing aggression and violence. However, despite its effectiveness, clozapine requires careful monitoring due to its potential side effects. Regular monitoring according to established guidelines is essential to manage the risks associated with clozapine therapy.

## 5. Conclusion

Our experience with low-dose clozapine treatment in patients with dual diagnoses at a low-security rehabilitation center demonstrated significant benefits, including improved engaged behaviors with staff and increased engagement in therapeutic sessions. These findings suggest that low-dose clozapine may be an effective intervention for managing aggression and behavioral issues in this population, adding to existing literature and supporting its use by clinicians. However, further research is necessary to fully explore the efficacy of clozapine and other antipsychotics in patients with personality disorders, especially within high-risk and dual-diagnosis subgroups.

## Figures and Tables

**Table 1:** Interpretation of DASA scores and corresponding risk levels.

Score	Level of risk	Action required
0–1	Low	No remedial action is required.

2–3	Moderate	The patient should be monitored for additional indicators of inpatient risk. Staff should be alerted to the possibility that the patient will become more agitated. Preventive measures should be considered.

>3	High	Remedial action is required. Staff must be alerted and the patient requires some remediation to prevent subsequent aggression from occurring. A risk management plan is required.

*Note: Source:* Royal College of Psychiatrists (n.d.). DASA Information [2].

Abbreviation: DASA, Dynamic Appraisal of Situational Aggression.

**Table 2:** Summary of demographics, substances abused, concomitant treatments, and 3-month outcomes for Cases A and B.

Parameter	Patient A	Patient B
Age (years)/gender	34/woman	30/man
Primary substances abused	Ketamine, pregabalin, diazepam	Alcohol, methamphetamine, cannabis
Concomitant treatments	Clozapine, aripiprazole, propranolol	Clozapine (monotherapy at discharge)
3-month outcome	Discharged; abstinent; returned to work	Discharged; attended one outpatient visit before disengagement

## Data Availability

The data that support the findings of this study are available upon request from the corresponding author. The data are not publicly available due to privacy or ethical restrictions.
